# Reverse Zoonotic Disease Transmission (Zooanthroponosis): A Systematic Review of Seldom-Documented Human Biological Threats to Animals

**DOI:** 10.1371/journal.pone.0089055

**Published:** 2014-02-28

**Authors:** Ali M. Messenger, Amber N. Barnes, Gregory C. Gray

**Affiliations:** 1 College of Public Health and Health Professions, University of Florida, Gainesville, Florida, United States of America; 2 Emerging Pathogens Institute, University of Florida, Gainesville, Florida, United States of America; Metabiota, United States of America

## Abstract

**Background:**

Research regarding zoonotic diseases often focuses on infectious diseases animals have given to humans. However, an increasing number of reports indicate that humans are transmitting pathogens to animals. Recent examples include methicillin-resistant *Staphylococcus aureus*, influenza A virus, *Cryptosporidium parvum*, and *Ascaris lumbricoides*. The aim of this review was to provide an overview of published literature regarding reverse zoonoses and highlight the need for future work in this area.

**Methods:**

An initial broad literature review yielded 4763 titles, of which 4704 were excluded as not meeting inclusion criteria. After careful screening, 56 articles (from 56 countries over three decades) with documented human-to-animal disease transmission were included in this report.

**Findings:**

In these publications, 21 (38%) pathogens studied were bacterial, 16 (29%) were viral, 12 (21%) were parasitic, and 7 (13%) were fungal, other, or involved multiple pathogens. Effected animals included wildlife (n = 28, 50%), livestock (n = 24, 43%), companion animals (n = 13, 23%), and various other animals or animals not explicitly mentioned (n = 2, 4%). Published reports of reverse zoonoses transmission occurred in every continent except Antarctica therefore indicating a worldwide disease threat.

**Interpretation:**

As we see a global increase in industrial animal production, the rapid movement of humans and animals, and the habitats of humans and wild animals intertwining with great complexity, the future promises more opportunities for humans to cause reverse zoonoses. Scientific research must be conducted in this area to provide a richer understanding of emerging and reemerging disease threats. As a result, multidisciplinary approaches such as One Health will be needed to mitigate these problems.

## Introduction

With today's rapid transport systems, modern public health problems are growing increasingly complex. A pathogen that emerges today in one country can easily be transported unnoticed in people, animals, plants, or food products to distant parts of the world in less than 24 hours [1]. This high level of mobility makes tracking and designing interventions against emerging pathogens exceedingly difficult, requiring close international and interdisciplinary collaborations. Fundamental to these efforts is an understanding of the ecology of emerging diseases. Published works often cite the large proportion of human emerging pathogens that originate in animals [2], [3], [4], [5]. However, scientific reports seldom mention human contributions to the variety of emerging diseases that impact animals. The focus of this review is to examine and summarize the scientific literature regarding such zoonoses transmission. A comprehensive table of the results is included in this document.

## Methods

For the purpose of this review several terms require definitions. Despite the fact that the term “zoonosis” usually refers to a disease that is transmitted from animals to humans (also called “anthropozoonosis”) [6], in this paper, “zoonosis” was defined as any disease that is transmitted from animals to humans, or vice versa [6], There are two related terms (“zooanthroponosis” and “reverse zoonosis”) that refer to any pathogen normally reservoired in humans that can be transmitted to other vertebrates [6]. Acknowledging that the terms “reverse zoonosis” or “zooanthroponosis” are seldom used, and that the term “zoonosis” can have several meanings, search methods were designed to include all of these terms in an effort to capture the widest possible subset of publications with documented human-to-animal transmission.

### Literature search

In June 2012, we searched PubMed in addition to several databases within Web of Knowledge and ProQuest to find articles documenting reverse zoonoses transmission. Search terms included: *reverse zoonosis*, *bidirectional zoonosis*, *anthroponosis*, *zooanthroponosis*, *anthropozoonosis*, and *human-to-animal disease transmission*. Articles were limited to clinical and observational type studies and were restricted to English only. Review articles were not included as they did not demonstrate a specific account of transmission. Letters to editors or similar correspondence were also excluded. Only publications with documented human-to-animal transmission were included. No time period was stipulated.

Four search strings were used for the PubMed database: ((bidirectional OR reverse) AND (zoono* or “disease transmission”)) OR anthropono* OR “human-to-animal”), ((bidirectional OR reverse OR “human-to-animal”) AND (zoono* or “disease transmission”)) OR anthropono*), (“reverse zoonoses” OR “ bidirectional zoonoses” OR “reverse zoonosis” OR “ bidirectional zoonosis” OR “reverse zoonotic” OR “ bidirectional zoonotic” OR anthropono* OR (“human-to-animal” AND disease* AND transmi*)), and (((bidirectional OR reverse OR “human-to-animal”) AND (zoonoses[majr] OR “Disease Transmission, Infectious”[majr] OR zoonosis[tiab] OR zoonoses[tiab] OR zoonotic[tiab])) OR Anthroponos*[tiab] OR Zooanthroponos*[tiab] OR Anthropozoonos*[tiab]). In the ProQuest and Web of Knowledge databases, we only used one string: ((bidirectional OR reverse) AND (zoonosis OR zoonoses OR zoonotic)) OR anthropono* OR Zooanthropono* OR anthropozoono* OR “human-to-animal” OR “human to animal”). The lack of additional search strings for the latter databases was due to less comprehensive search capabilities. Duplicate articles were removed.

### Literature analyses

Titles and abstracts were reviewed and articles were retained when there was evidence of disease transmission from humans to animals. During full text review, some citations proved straightforward in distinguishing transmission from humans to animals (e.g. via direct contact), while others were selected based on strong author suggestion or research implications toward reverse zoonotic transmission. In an effort to highlight trends in an otherwise diverse set of articles, citations were grouped by pathogen type and year of publication. To further clarify relationships, we also pictorially displayed the study locations and animal types discussed in the various articles.

## Results

**Figure 1:**
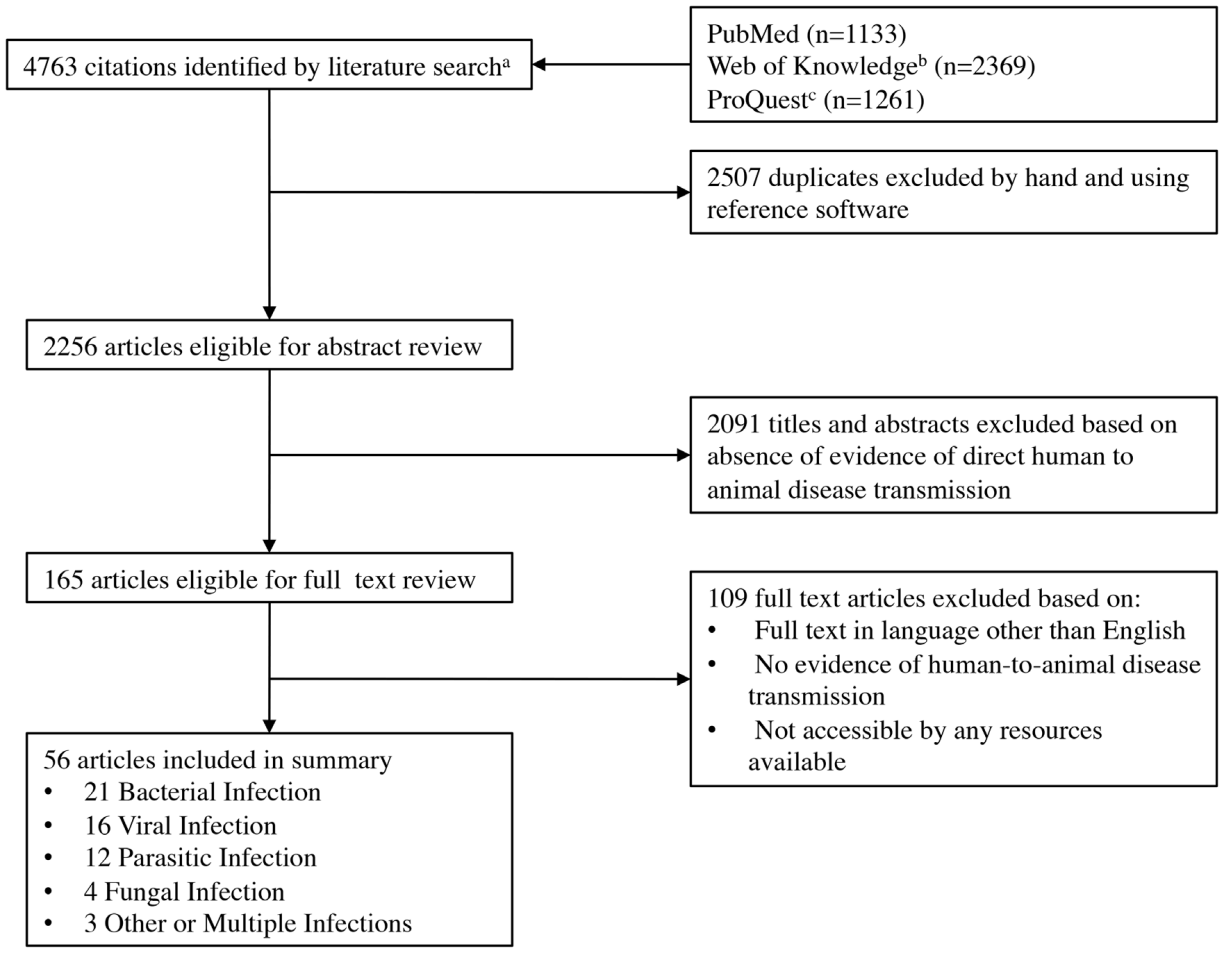
Flowchart demonstrating the identification and selection process for publications included in this review.

This comprehensive literature review yielded 4763 titles, 2507 of which were excluded as duplicates (Figure 1). During the review of abstracts, 2091 studies were excluded due to a lack of evidence of human-to-animal disease transmission. After consideration of the 165 eligible for full text review, 109 studies were excluded based on full texts being written in a language other than English, absence of human-to-animal disease transmission, or full texts being unavailable. After all exclusions, 56 articles were considered for this review (Table 1).

**Table 1:** Descriptors of reports included in review with documented human-to-animal transmission.

Publications	Study Location	Specimen Source	Pathogen Name	Animal(s) Infected
**Bacteria**				
Cosivi et al (1995) [7]	Morocco	Assorted	*Mycobacterium tuberculosis, Mycobacterium bovis* 1	Wildlife
Seguin et al (1999) [8]	United States	Veterinary hospital	*Methicillin-resistant Staphylococcus aureus* (MRSA)^1^^,^ 2	Livestock
Donnelly et al (2000) [9]	United States	4H project livestock	*Streptococcus pneumonia* 1	Livestock
Nizeyi et al (2001) [10]	Uganda	National park	*Campylobacter* spp., *Salmonella* spp., *Shigella sonnei*, *Shigella boydii*, *Shigella flexneri* 1 ^,^ 3	Wildlife
Michel et al (2003) [11]	South Africa	Zoo	*M. tuberculosis* 1 ^,^ 3	Wildlife
Hackendahl et al (2004) [12]; also see Erwin et al (2004) [13]	United States	Veterinary hospital	*M. tuberculosis* 1 ^,^ 4	Companion
Prasad et al (2005) [14]	India	Veterinary hospital	*M. tuberculosis* 3 ^,^ 4	Livestock
Weese et al (2006) [15]	Canada, United States	Household; Veterinary hospital	MRSA^1^	Companion
Morris et al (2006) [16]	United States	Household; Veterinary hospital	MRSA^1^	Companion
Kwon et al (2006) [17]	Korea	Slaughterhouse	MRSA^1^^,^ 3	Companion; Livestock
Rwego et al (2008) [18]	Uganda	National park	*Escherichia coli* 1 ^,^ 3	Livestock; Wildlife
Hsieh et al (2008) [19]	Taiwan	Livestock farm	*Oxacillin-resistant Staphylococcus aureus* (ORSA)	Livestock
Berg et al (2009) [20]	Ethiopia	Slaughterhouse	*M. tuberculosis* 3	Livestock
Heller et al (2010) [21]	United Kingdom	Household; Veterinary hospital	MRSA^1^^,^ 2	Companion
Kottler et al (2010) [22]	United States	Household; Veterinary hospital	MRSA^1^	Companion
Ewers et al (2010) [23]	Germany, Italy, Netherlands, France, Spain, Denmark, Austria & Luxembourg	Veterinary hospital	*Escherichia coli*	Companion; Livestock
Every et al (2011) [24]	Australia	University zoology department	*Helicobacter pylori* 1	Wildlife
Lin et al (2011) [25]	United States	Veterinary hospital	MRSA^1^	Companion; Livestock
Rubin et al (2011) [26]	Canada	Veterinary hospital; Human hospital	MRSA^1^	Companion
Price et al (2012) [27]	Austria, Belgium, Canada, Switzerland, China, Germany, Denmark, Spain, Finland, France, French Guiana, Hungary, Italy, the Netherlands, Peru, Poland, Portugal, Slovenia, and United States	Animal meat for sale	MRSA^1^	Livestock
**Virus**				
Meng et al (1998) [28]	United States	Veterinary laboratory; Human sample	Hepatitis E^5^	Wildlife
Willy et al (1999) [29]	United States	Veterinary laboratory	Measles^1^^,^ 4	Wildlife
Kaur et al (2008) [30]	Tanzania	National park	Human metapneumovirus (hMPV)^1^^,^ 4	Wildlife
Feagins et al (2008) [31]	United States	Commercially sold laboratory animals	Hepatitis E^5^	Livestock
Song et al (2010) [32]	South Korea	Livestock farm	Influenza A (2009 pandemic H1N1)^1^	Livestock
Swenson et al (2010) [33]	United States	Household; Veterinary hospital	Influenza A (2009 pandemic H1N1)^1^^,^ 4	Companion
Tischer et al (2010) [34]	Various; Unspecified	Unknown (previous reports cited)	Human herpesvirus 1, human herpesvirus 4^1^^,^ 3 ^,^ 4	Companion; Wildlife
Abe et al (2010) [35]	Japan	Wildlife	Rotavirus^1^^,^ 3	Wildlife
Berhane et al (2010) [36]	Canada, Chile	Livestock farm	Influenza A (2009 pandemic H1N1)^1^^,^ 4 ^,^ 5	Livestock
Poon et al (2010) [37]	Hong Kong	Slaughterhouse	Influenza A (2009 pandemic H1N1)	Livestock
Forgie et al (2011) [38]	Canada	Veterinary laboratory	Influenza A (2009 pandemic H1N1)	Livestock
Holyoake et al (2011) [39]	Australia	Livestock farm	Influenza A (2009 pandemic H1N1)^4^	Livestock
Scotch et al (2011) [40]	Mexico, United States, Canada, Australia, United Kingdom, France, Ireland, Argentina, Chile, Singapore, Norway, China, Italy, Thailand, South Korea, Indonesia, Germany, Japan, Russia, Finland, and Iceland	Unknown (previous reports cited)	Influenza A (2009 pandemic H1N1)	Companion; Livestock; Wildlife
Trevennec et al (2011) [41]	Vietnam	Livestock farm; Slaughterhouse	Influenza A (2009 pandemic H1N1)^1^^,^ 2	Livestock
Wevers et al (2011) [42]	Cameroon, Democratic Republic of the Congo, Gamiba, Côte d'Ivoire, Republic of Congo, Rwanda, Tanzania, Uganda, Germany (initial samples in Asia and South America)	Wildlife; Zoo	Human adenovirus A-F^1^^,^ 3	Wildlife
Crossley et al (2012) [43]	United States	Private zoo	Influenza A (2009 pandemic H1N1)^2^^,^ 3	Wildlife
**Parasite**				
Sleeman et al (2000) [44]	Rwanda	National park	*Chilomastix mesnili, Endolimax nana, Stronglyoides fuelleborni, Trichuris trichiura* 1 ^,^ 3	Wildlife
Graczyk et al (2001) [45]	Uganda	National park	*Cryptosporidium parvum*	Wildlife
Graczyk et al (2002) [46]	Uganda	National park	*Encephalitozoon intestinalis* 1 ^,^ 3	Wildlife
Graczyk et al (2002) [47]	Uganda	National park	*Giardia duodenalis* 1 ^,^ 3	Wildlife
Guk et al (2004) [48]	Korea	Laboratory	*C. parvum* 5	Livestock; Wildlife
Noël et al (2005) [49]	Singapore, Pakistan, Japan, Thailand, United States, France, Czech Republic	N/A	*Blastocystis* spp	Livestock; Wildlife
Coklin et al (2007) [50]	Canada	Livestock farm	*G. duodenalis, C. parvum* 1 ^,^ 3	Livestock
Adejinmi et al (2008) [51]	Nigeria	Zoo	*Ascaris lumbricoides, T. trichiura* 1	Wildlife
Teichroeb et al (2009) [52]	Ghana	Wildlife	*Isospora* spp., *Giardia duodenalis* 1 ^,^ 3	Wildlife
Ash et al (2010) [53]	Zambia; Namibia; Australia	Wildlife; Zoo	*G. duodenalis* 1	Wildlife
Johnston et al (2010) [54]	Uganda	National park	*G. duodenalis* 1 ^,^ 3	Livestock; Wildlife
Dixon et al (2011) [55]	Canada	Livestock farm	*G. duodenalis, C. parvum* 1 ^,^ 3	Livestock
**Fungus**				
Jacobs et al (1988) [56]	Unspecified	Assorted	Microsporum spp., Trichophyton spp.^1^	Assorted
Pal et al (1997) [57]	India	Household	*Trichophyton rubrum* 1	Wildlife
Wrobel (2008) [58]	United States	Veterinary hospital	*Candida albicans* 3	Companion; Livestock; Wildlife
Sharma et al (2009) [59]	India	Household; Veterinary hospital	*Microsporum gypseum* 1	Wildlife
**Other**				
Epstein et al (2009) [60] +	Assorted	Wildlife; Livestock farm; Zoo; Laboratory	Herpes simplex 1, influenza A, parasite spp, Measles, MRSA, *M. tuberculosis* 1 ^,^ 2 ^,^ 3 ^,^ 4 ^,^ 6	Assorted
Guyader et al (2000) [61] &	France	Shellfish-growing waters	Astrovirus, enterovirus, hepatitis A, Norwalk-like (norovirus), rotavirus^1^^,^ 3	Wildlife
Muehlenbein et al (2010) [62] ∧	Malaysia	Wildlife	Assorted illnesses^1^^,^ 6	Wildlife

**Other assorted pathogen types:**

+ **virus; parasite/bacteria,**

& **virus/bacteria,**

∧ **assorted.**

**Modes of transmission as indicated by authors:**

1 **direct contact,**

2 **fomite,**

3 **oral,**

4 **aerosol,**

5 **inoculation,**

6 **other.**

**Figure 2:**
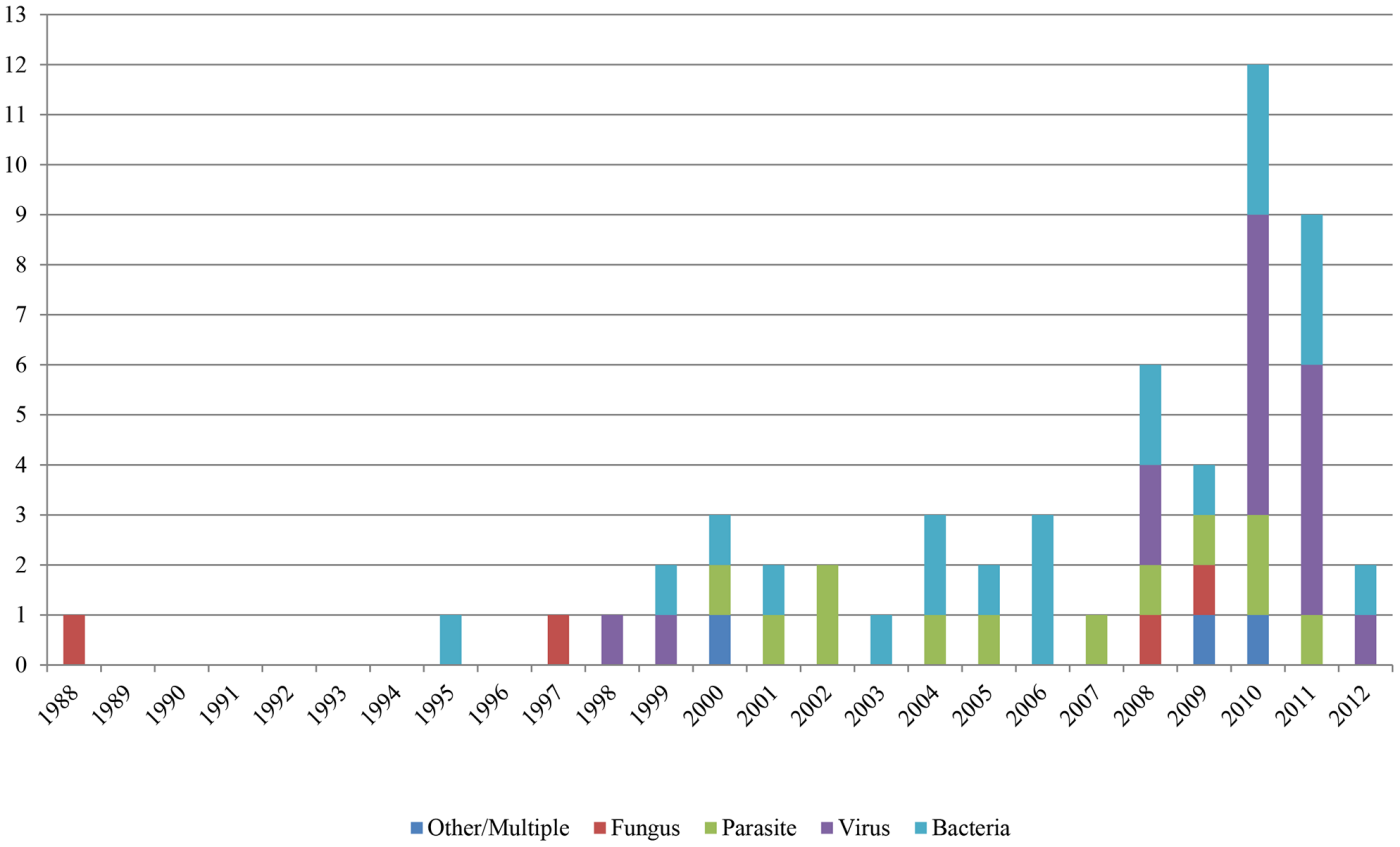
Timeline and frequency of reverse zoonoses publications included in this review shown by pathogen type.

**Figure 3:**
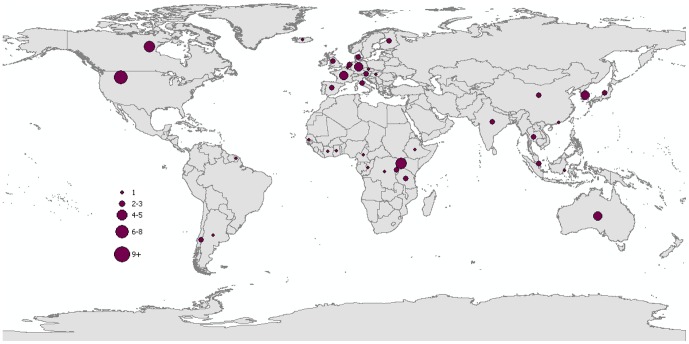
Proportion of reverse zoonoses scientific reports included in review as illustrated by study location. Note: Many reports identified several countries therefore each country in this figure does not necessarily represent a single corresponding publication.

**Figure 4:**
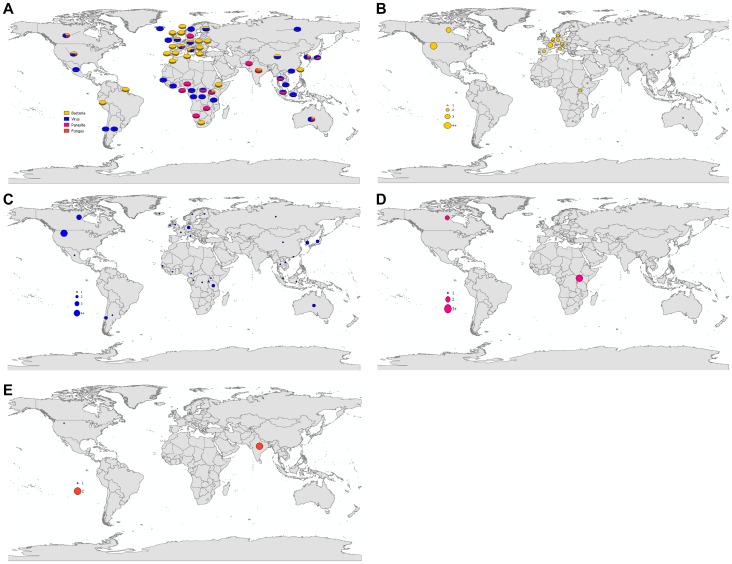
Study locations for literature included in review. A. Proportion of reverse zoonoses scientific reports as illustrated by study location and pathogen type; B. Proportion of reverse zoonoses scientific reports on bacterial pathogens as illustrated by study location; C. Proportion of reverse zoonoses scientific reports on viral pathogens as illustrated by study location; D. Proportion of reverse zoonoses scientific reports on parasitic pathogens as illustrated by study location; E. Proportion of reverse zoonoses scientific reports on fungal pathogens as illustrated by study location.

Included reports were based in 56 different countries. Although the reports spanned three decades, there seems to be an increasing number of studies published in recent years (Figure 2). Twenty eight percent of the studies were conducted in the United States (n = 16), 14% in Canada (n = 8), and 13% in Uganda (n = 7) (Figure 3). Within the study results, 21 publications discussed human-to-animal transmission of bacterial pathogens (38%); 16 studies discussed viral pathogens (29%); 12 studies discussed human parasites (21%); and seven studies discussed transmission of fungi, other pathogens, or diseases of multiple etiologies (13%). Bacterial pathogen reports were centered in North America and Europe. Viral studies were well-distributed globally. Parasitic disease reports were conducted chiefly in Africa. Fungal studies were conducted almost exclusively in India (Figure 4).

**Figure 5:**
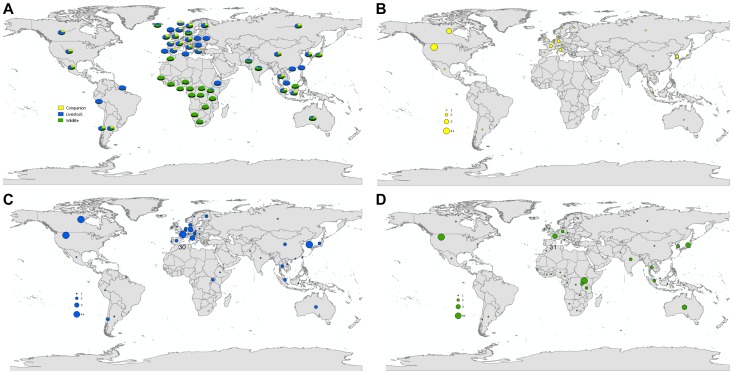
Animal type and study location included in review literature. A. Proportion of reverse zoonoses scientific reports as illustrated by study location and animal(s) infected; B. Proportion of reverse zoonoses scientific reports on companion animals as illustrated by study location; C. Proportion of reverse zoonoses scientific reports on livestock as illustrated by study location; D. Proportion of reverse zoonoses scientific reports on wildlife as illustrated by study location.

Animals with reported infection or inoculation with human diseases included wildlife (n = 28, 50%), livestock (n = 24, 43%), companion animals (n = 13, 23%), and other animals or animals not explicitly mentioned (n = 2, 4%). The majority of companion and livestock animals were studied in North America and Europe, while wildlife studies were most prevalent in Africa (Table 1, Figure 5). Typically, diagnostic specimens were collected at veterinary hospitals (n = 15, 27%), national parks (n = 8, 14%) and livestock farms (n = 8, 14%). Direct contact was the suggested transmission route 71% of the time (n = 40). Other transmission routes included fomite, oral contact, aerosols, and inoculation.

As early as 1988, zoonoses research focusing on fungal pathogens was being conducted. Initial studies implied human transmission of *Microsporum* (n = 2) and *Trichophyton* (n = 2) to various animal species, with a later article centered on *Candida albicans* (n = 1) (Figure 2). These publications were set in India (n = 2) and the United States (n = 1).

Since 1988, research with implications of reverse zoonoses has been largely focused on infections of bacterial origin, beginning in 1995. The majority of articles in this review focused on methicillin-resistant *Staphylococcus aureus* (MRSA) (n = 9) and *Mycobacterium tuberculosis* (n = 5). Reports regarding these bacteria were primarily conducted in the United States (n = 8) among livestock (n = 10) or companion animals (n = 9).

Viruses were the second most common pathogen associated with human-to-animal transmission. Reverse zoonoses reports regarding viral pathogens began in 1998 and have since been focused primarily on influenza with great interest surrounding the 2009 H1N1 pandemic (n = 9). These studies were conducted largely in the United States (n = 6) in livestock (n = 8) and wildlife (n = 8).

Studies suggestive of transmission of human parasites to animals were first published in 2000. The most commonly reported parasitic agents to be transmitted from humans to animals were *Giardia duodenalis* (n = 6) and *Cryptosporidium parvum* (n = 4). Parasitic research has been carried out most frequently in Uganda (n = 4) and Canada (n = 2). The authors investigated human parasitic infections chiefly in wildlife (n = 10) and livestock (n = 5).

Human-to-animal transmission is plausible for a large number of diseases because the pathogens concerned are known to infect multiple species [3]. For instance, 77.3% of the pathogens infecting livestock are considered “multiple species pathogens [3].” However, this review only found 24 reports which considered reverse zoonoses disease transmission as a potential threat to livestock, underscoring a need for further research in this area [3]. Similarly, in companion animals this review found even fewer studies (n = 13) that implied reverse zoonoses as a possible cause of infection, despite the fact that 90% of known pathogens for domestic carnivores are recognized as “multiple species pathogens [3].” The majority of publications in this reverse zoonoses review involved studies documenting human-to-wildlife transmission (n = 28). Unfortunately, they too were severely lacking in comparison to the research need. Each type of animal- livestock, companion, or wildlife, represents a unique set of risk factors for reverse zoonoses through their specific routes of human contact.

## Discussion

Human and animal relationships are likely to continue to intensify worldwide over the next several decades due in part to animal husbandry practices, the growth of the companion animal market, climate change and ecosystem disruption, anthropogenic development of habitats, and global travel and commerce [2]. As the human-animal connection escalates, so does the threat for pathogen spread [1], [63]. This review notes a number of factors that influence the risk of disease transmission from humans to animals.

For instance, human population growth and expansion encourages different species to interact in ways and at rates previously not encountered, and to do so in novel geographical areas [4]. The term “pathogen pollution” refers to the process of bringing a foreign disease into a new locality due to human involvement [64]. In the case of the endangered African painted dog, wild dogs have been infected with human strains of *Giardia duodenalis*, leading researchers to believe that pathogen pollution occurred through open defecation in and around national parks by tourists and local residents [53]. Anthropogenic changes in the ecosystem increase the amount of shared habitats between humans and animals thus exposing both to new pathogens. Researchers discovered the human strain of pandemic *Escherichia coli* strain 025:H4-ST131 CTX-M-15 in many different species of animals indicating inter-species transmission from humans to pets and livestock [23]. This particular human strain found to be infecting animals was documented across Europe.

In addition to habitat change, growth, and/or destruction, there is the ever-increasing global movement of products and travelers that extends to both humans and animals. During the pandemic of 2009 H1N1 influenza, the novel virus was able to travel across the globe and from humans to swine in less than two months [32]. One driving force behind the movement of animals and animal products is the worldwide shipment of meat. This phenomenon is a relatively new event as developing countries adjust their diets to include more meat- and dairy-based products [4]. While food and animal safety guidelines attempt to keep up with the speed of global trade, international efforts appear to be outpaced by product demand. For example, it has been estimated that five tons of illegal bushmeat pass through Paris' main Roissy-Charles de Gaulle airport each week in personal luggage [65]. However, overt retail systems of animal and animal products can also contribute to the danger of zoonoses and reverse zoonoses transmission. Many animals are sold in markets which allow humans and a myriad of animal species to interact in conditions that are known to trigger emerging diseases [66]. Specifically, this is true for live animal markets and warehouses for exotic pets [4].

The pet industry is an enormous global business that now expands from domestic to exotic animals. A 2011–2012 national pet owners survey found that in the United States alone, 72.9 million homes or 62% of the population have a pet [67]. Of these pets, the majority of animals are dogs (78.2 million) or cats (86.4 million), but a large number of pets are birds (16.2 million), reptiles (13 million), or small animals (16 million) [67]. As pet ownership seems to be increasing worldwide and more exotic pets are being introduced to private homes, the potential for disease transmission between humans and animals will continue to increase. Veterinarians must more fervently protect animals under their care from human disease threats [68]. Adopting a One Health strategy for emerging disease surveillance and reporting will benefit both humans and animals and produce a more collaborative response plan.

Veterinarians, animal health workers, and public health professionals are not the only ones who should recognize the threat of reverse zoonoses. Increased awareness must also be communicated to the general public. Worldwide, there are 1,300 zoos and aquariums that sustain more than 700 million visitors each year [69]. The potential for pathogen spread to animals can come from a visitor with an illness, contamination of a shared environment or food, and the spread of disease through relocation of animals for captivity or educational purposes. In Tanzania, a fatal outbreak of human metapneumovirus in wild chimpanzees is believed to be the result of researchers and visitors viewing the animals in a national park that was once the great apes' territory [30]. Public education and awareness should be augmented to include the potential health threats inflicted on a susceptible animal by an unhealthy human.

This report has limitations. As demonstrated in this review paper, the trend for reporting pathogen spread of human-to-animal is increasing. However the route of human transmission to animal disease manifestation is often unknown in these reports and not well documented in this review. Also the report did not examine articles that did not document human-to-animal transmission. We acknowledge that many additional works that have recorded the existence of human pathogens in animals were not evaluated. However, this review was designed to summarize only the publications that document reverse zoonotic transmission.

Many common and dangerous pathogens have not, to the authors' knowledge, been researched as reverse zoonoses threats to animals representing a significant gap in the scientific literature. Future investigations of reverse zoonoses should take into account both transmission routes and disease prevalence. Prospective research should also include a wider variety of etiological agents and animal species. Scientific literature must document the presence and transmission of human diseases in animals such that the wealth of literature on this subject will become defined and accessible across multiple disciplines. A wider knowledge and understanding of reverse zoonoses should be sought for a successful One Health response. We recommend that future research be conducted on how human disease can, and does, affect the animals around us.

## Supporting Information

File S1 **PLOS PRISMA 2009 checklist.**
(DOC)
